# The Role of Distorted Cognitions in Mediating Treatment Outcome in Children with Social Anxiety Disorder: A Preliminary Study

**DOI:** 10.1007/s10578-021-01268-6

**Published:** 2021-10-21

**Authors:** Lynn Mobach, Ronald M. Rapee, Anke M. Klein

**Affiliations:** 1 Centre for Emotional Health, Department of Psychology, Macquarie University, Sydney, NSW 2109 Australia; 2 Present Address: Department of Clinical Psychology, Behavioural Science Institute, Radboud University, Montessorilaan 3, 6525 HR Nijmegen, the Netherlands; 3 Unit Developmental and Educational Psychology, Institute of Psychology, Leiden University, Leiden, the Netherlands

**Keywords:** Social anxiety disorder, Cognitive-behavioural therapy (CBT), Distorted cognition, Interpretation, Negative beliefs

## Abstract

This study examined whether distorted cognition changes during cognitive behavioural therapy (CBT) in children (*N* = 61; aged 7–12) with social anxiety disorder (SAD) and whether changes in distorted cognition from pre- to post-treatment predict SAD at 6-month follow-up. Baseline distorted cognition was also examined as a predictor of post-treatment outcome. Multiple informant SAD-measures were obtained pre-treatment, post-treatment and at 6-month follow-up. Children reported on interpretation bias and dysfunctional beliefs. A decrease in interpretation bias and dysfunctional beliefs was prospectively related to greater SAD change between post-treatment and 6-month follow-up. Child-reported SAD-change at post-treatment predicted greater change in dysfunctional beliefs at 6-month follow-up. Higher baseline interpretation bias predicted greater change in SAD-severity at post-treatment. Children with greater distorted cognition reductions during treatment, showed greater treatment gains at 6-month follow-up. Children who do not show this reduction may require additional efforts focused on distorted cognition to maximally benefit from treatment.

**Supplementary Information:**

The online version contains supplementary material available at 10.1007/s10578-021-01268-6.

About 60% of children with an anxiety disorder remit after receiving cognitive behavioural therapy (CBT) [1]. Children with social anxiety disorder (SAD) anywhere in their diagnostic profile are more likely than other anxious youth to continue to meet diagnostic criteria and to show residual anxiety symptoms following treatment compared to children without SAD [2–4]. Childhood SAD is associated with severe disruptions in social, family and academic functioning and a higher risk for development of other disorders [5, 6]. Understanding treatment mediators and baseline predictors of change for childhood SAD can help to determine how treatment works and for whom treatment works. This can indicate specificity of treatment effects and subsequently aid in refining treatment for children who do not benefit from current approaches.

Theoretical underpinnings of anxiety disorders suggest that cognitive distortions are central to the development and maintenance of SAD [7–9]. Indeed, a considerable number of studies indicate that children with SAD negatively interpret ambiguous situations (i.e., interpretation bias) and that they hold negative assumptions and beliefs about their functioning and their ability to cope with perceived threats [10–13]. In line with theory, for anxiety symptoms to change, changes in distorted cognition should occur. The principal components of CBT propose to do just that: Exposure to feared stimuli or situations and cognitive techniques should help anxious children to identify and correct cognitive distortions. This process is assumed to underpin positive changes in anxiety symptoms.

CBT is indeed effective in producing positive changes in child anxiety [1]. However, the exact mechanism through which CBT exerts its effects is still largely unclear [14]. Researchers have started to investigate mediators of CBT for childhood anxiety disorders [15–19]. Surprisingly, despite the theoretical assumptions of CBT [7, 20], only a handful of studies has investigated cognitive variables as potential mediators. Furthermore, although consistent baseline predictors of treatment outcome are scarce [21], few studies have looked into baseline levels of distorted cognition as potential treatment outcome predictors [22]. The current study will therefore focus on cognitive change throughout treatment as a potential mediator to examine how treatment works and on baseline levels of cognitive distortions as treatment outcome predictors to understand for whom treatment works.

Studies that have examined cognitive change during child anxiety treatment, showed mixed results. Consistent with theory, studies have shown that a change in distorted cognition is associated with symptom change [17, 18, 23, 24]. However, some studies did not (or only partially) demonstrate this association [16, 19, 25]. To our best knowledge, there are no studies that have specifically focused on cognitive change during treatment for childhood SAD. This limits the conclusions that can be drawn for this group. Given that children with SAD have poorer treatment outcomes than other anxious children, it is crucial that studies focus on childhood SAD to investigate factors that may underlie this poorer treatment response.

With regards to how treatment works, in one study, Waters et al. [24] found that in a sample of children with mixed anxiety disorders (aged 8–12), interpretation bias significantly decreased from pre- to post-CBT. Residual threat interpretations were associated with higher anxiety symptoms in anxious versus non-anxious children. Also, in a sample of children (aged 8–13) with elevated anxiety levels, gains after CBT on child self-reported anxiety symptoms were significantly associated with a decrease in dysfunctional beliefs [23]. In contrast, another study found that although youth (aged 7–17) benefitted from treatment and the introduction of cognitive restructuring accelerated progress, this could not be attributed to changes in dysfunctional beliefs [19]. These studies provide important initial information on possible cognitive mediators of change during CBT for child anxiety in general. Primarily, the results seem to indicate that different forms of cognition may respond differently to treatment. However, conclusions are inherently limited due to their correlational nature and focus on post-treatment outcome which introduces the possibility that shared method variance may have (partially) explained the associations between outcome measures [23, 24].

Somewhat stronger evidence for mediation comes from two studies incorporating a treatment control group. Treadwell and Kendall [26] showed that children’s change in dysfunctional beliefs predicted change in self-reported (but not parent- or teacher-reported) anxiety throughout treatment. In a similar vein, Kendall and Treadwell [27] concluded that changes in dysfunctional beliefs mediated treatment gains. These studies only included pre- and post-treatment assessments, prohibiting examination of mediation of post-treatment or 6-month follow-up treatment outcomes or conclusions on temporal precedence, as there were less than three timepoints. However, these studies indicate an association between changes in distorted cognition and changes in anxiety.

Studies that include more than two assessments provide stronger evidence for cognitive change as a mediator of treatment outcome. A recent study included pre-, in- and post-treatment, and 4-month follow-up assessments [18]. The authors found support for the role of an interpretation bias-reduction in explaining a significant proportion of the variance in anxiety symptoms at post-treatment and 4-months follow-up in a non-treatment seeking sample of children with anxiety disorders (aged 8–12). Similarly, another study found evidence for cognitive change as a mediator of 6-month follow-up treatment outcomes in a treatment-seeking sample of children with anxiety disorders (aged 7–12) [17]. The results indicated that changes in dysfunctional beliefs and maladaptive metacognitions predicted post-treatment anxiety. However, only changes in dysfunctional beliefs were related to child-self reported anxiety symptoms at 6-month follow-up. In a similar study, treatment-seeking youth (8–18 years) with an anxiety disorder were assessed at pre-, in-, and post-treatment and at 3-month follow-up [25]. The authors examined a range of possible mediators and found amongst others that an increase in positive thoughts preceded changes in child-reported anxiety symptoms. However, there was no evidence that a decrease in dysfunctional beliefs mediated treatment outcome. In line with this study, Kendall et al. [16] also failed to find evidence for the role of decreased dysfunctional beliefs as a mediator of anxiety reduction at 3-month follow-up in a treatment-seeking sample with mixed anxiety disorders (aged 7–17).

In sum, the majority of the studies on mediators of child anxiety treatment have shown that CBT impacts several distorted cognition indices and that there is an association between change in distorted cognition and treatment outcome. This seems promising in light of the underlying CBT-framework. However, there are also several studies that do not find this relation. Previous studies included widely varying designs and combined different distorted cognition indices with different informants to report on outcome variables. Currently, these studies do not allow for clear conclusions on which forms of cognition are effectively targeted by CBT, and which cognition types need to change for a decrease in anxiety symptoms to occur. Furthermore, the previous focus on mixed anxiety does not provide any indication of possible reasons for the poor treatment response among children with SAD. Therefore, it is pivotal to investigate changes in different distorted cognition types and their relation to social anxiety symptoms measured by multiple-informant reports [12].

In this study, the focus lies on interpretation bias and dysfunctional beliefs among socially anxious youth: Interpretation bias has been pinpointed as a possible treatment mediator across anxiety disorders [18], but has been understudied and results [18] need to be replicated. Also, previous studies found mixed results with regard to the role of changes in dysfunctional beliefs [25, 27]. Importantly, both of these cognitive constructs have been linked to the maintenance of childhood social anxiety [10, 11, 13]. Furthermore, given the differential effectiveness of CBT for children with SAD versus children with non-social anxiety disorders, investigating heterogeneous anxiety disorder groups may lead to obscured results. The high comorbidity between anxiety disorders is often given as the reason to study this group together. However, despite these high comorbidity rates, unique impairments exist [28, 29]. Therefore, it is necessary to investigate disorder-specific treatment mediators to gain more insight into the mediating role of cognitive change in obtaining beneficial treatment outcome in children with SAD. Additionally, the identification of baseline treatment-outcome predictors can provide important insights into for whom treatment works. Surprisingly, not many studies have examined cognitive treatment-outcome predictors in children with SAD. An exception is a study that indicates that anxious children with higher negative attention bias levels (vs. those with low levels) at pre-treatment showed significantly more improvement on anxiety symptoms at post-treatment [22]. Clearly, identification of pre-treatment predictors for children with SAD and exploration of cognitive variables as pre-treatment predictors needs further attention.

The present study examines how and for whom treatment works best in a clinical treatment-seeking sample of children with SAD. The first aim was to examine how treatment works, by assessing (1a) whether children with SAD show pre- to post-treatment changes on two indexes of distorted cognition (interpretation bias and dysfunctional beliefs) and subsequently, whether (1b) pre- to post-treatment changes in cognition were related to social anxiety levels at 6-month follow-up. The second aim was to examine for whom treatment works best by assessing whether baseline levels of the cognitive variables predicted post-treatment outcome (aim 2). We included child-, parent- and clinician-report of social anxiety to gain a comprehensive understanding of social anxiety severity. It was hypothesized that children would show decreases in interpretation bias and dysfunctional beliefs from pre- to post-treatment [8, 9]. Also, with regards to the directionality of change, it was hypothesized that pre- to post-treatment cognitive changes would be prospectively related to SAD diagnostic severity and social anxiety symptoms at 6-month follow-up. More specifically, it was expected that children who showed greater decreases on the distorted cognition indices, would show greater decreases in SAD severity. The hypothesis regarding pre-treatment outcome predictors was exploratory in nature in light of the lack of studies in this area. This is the first study to explore potential treatment mechanisms and cognitive baseline predictors that may explain the lack of treatment response for children with SAD. This study therefore represents an important first step into unravelling possible reasons that childhood SAD predicts less favourable treatment outcomes.

## Methods

### Participants

Sixty-one children with SAD anywhere in their diagnostic profile participated in this study. Of these children, 26.2% had a primary (most interfering) SAD diagnosis and 73.8% had a secondary SAD diagnosis. Children were selected from a larger sample of children with primary anxiety disorders on the waiting list for generic anxiety treatment (see Table 1 for diagnostic profile characteristics). Children were aged 7–12 (*M* = 9.23, *SD* = 1.56; 41.0% girls). Children were recruited through the Centre for Emotional Health in Sydney, Australia. Exclusion criteria were high-risk suicidal ideation, concurrent psychological treatment, intellectual impairment, psychotic symptoms, and physical or sexual abuse in their home environment. Appropriate referrals were given to children and their families if any of these exclusion criteria were present. The current dataset partially overlapped with participant data that was used in two other studies on interpretation bias and the effect of Cognitive Bias Modification for interpretation bias (CBM-I)^1^ [10, 30]. The Human Research Ethics Review Committee of Macquarie University approved the current study.

**Table 1:** Prevalence (%) of diagnoses for primary and all secondary diagnoses

	Primary disorder %	Secondary disorder^a^ %
SAD	26.2	73.8
GAD	60.7	44.3
SEP	3.3	41.0
SP	4.9	95.1
OCD	4.9	9.8
Mood disorder	0	14.8
Externalising disorder	0	23.0

*SAD* social anxiety disorder; *GAD* generalised anxiety disorder; *SEP* specific phobia; *OCD* obsessive compulsive disorder

^a^Prevalence of all secondary diagnoses together

### Materials

#### Anxiety Disorders Interview Schedule for DSM-IV-Child/Parent (ADIS-IV-C/P) [31]

The ADIS-IV-C/P is a semi-structured clinical interview to diagnose anxiety disorders and other common disorders in youth according to the Diagnostic and Statistical Manual-fourth edition (DSM-IV; American Psychiatric Association [APA]) [32]. Interviews were held separately with the parent(s) and the child and all disorders were rated with a clinician severity rating (CSR; 0 [*no interference*] to 8 [*extreme interference*]). CSRs ≥ 4 indicate a clinically interfering disorder. In the current study, the composite diagnosis was used to determine diagnoses and CSRs for treatment evaluation. An overall interference rating (0 [*low daily functioning*] to 100 [*high daily functioning*]) was administered using the Children’s Global Assessment Scale (CGAS) [33]. The CGAS has shown acceptable psychometric properties [34].

#### Spence Children’s Anxiety Scale-Child/Parent (SCAS-C/P) [35, 36]

The SCAS-C/P assesses anxiety symptoms by child self-report and parent-report for the child across multiple anxiety domains. For this study, only the 6-item social scale of the SCAS was used. Items were answered on a 4-point Likert-scale (0 [*never*] to 3 [*always*]). Higher scores indicate higher social anxiety levels. The SCAS-social scale has previously demonstrated acceptable psychometric properties [35, 36]. Internal consistency in this study was acceptable (SCAS-C: pre-treatment *α* = 0.82, post-treatment *α* = 0.69, 6-month follow-up *α* = 0.66; SCAS-P: pre-treatment *α* = 0.79, post-treatment, *α* = 0.84, 6-month follow-up *α* = 0.82).

#### Children’s Automatic Thoughts Scale (CATS) [37]

The CATS assesses dysfunctional beliefs with 40 items with four scales, but in the current study only the social threat scale was used. Items are scored on a 5-point Likert scale (0 [*not at all*] to 4 [*all the time*]). Higher scores indicate higher levels of social threat thoughts. The CATS has demonstrated good psychometric properties in community and clinical samples [37, 38]. Internal consistency in the current study was good (pre-treatment *α* = 0.95, post-treatment *α* = 0.93, 6-month follow-up *α* = 0.91).

#### Interpretation Task [30]

The interpretation task consisted of 45 (social, physical, neutral) ambiguous scenarios to assess interpretation bias. The social threat scenarios were used in the current study. The scenarios were a mix of existing materials [30, 39–43]. Three random sets of 5 social scenarios were created to lessen the burden on the children. Children could choose from a positive, neutral/positive, neutral/negative, and negative ending. Scenarios were presented in a pseudo-randomized order, such that no more than two scenarios of the same category would be shown sequentially. Children were instructed to read the story aloud, to imagine themselves being the central character in the story and to choose the ending that they thought would suit the story best. Internal consistency of the social scenarios was acceptable (pre-treatment *α* = 0.66, post-treatment *α* = 0.65) and comparable to previous studies [10, 43].

### Procedure

Families seeking treatment of their child’s anxiety symptoms were invited to the clinic and went through a diagnostic assessment (ADIS-IV-C/P) [31, 32]. If children were diagnosed with a primary anxiety disorder, they were automatically allocated to generic CBT. While children were on the waiting list, they completed the CBM-I.^2^ Before CBT (and before CBM-I) started (T1), children filled out the interpretation task, the CATS and SCAS-C. Parents filled out the SCAS-P. All tasks and questionnaires were presented electronically on a computer screen. Within 2 weeks after the last CBT session (T2) and at 6-month follow-up (T3), the same procedure was repeated (except for the interpretation bias task at T3). Interviews were held by qualified clinical psychologists or trained clinical psychology graduate students under the supervision of a senior clinical psychologist.

### Treatment

The Cool Kids program is a manualized, structured, ten-session, CBT-program [44]. For the current study, sessions were 2 hours in length and conducted in groups of around 6–8 families. This program focuses on recognizing emotions, restructuring distorted cognitions, and gradual exposure to feared situations. Children and their parents are taught strategies and skills to manage the child’s anxiety. Active components of the Cool Kids program include psychoeducation, cognitive restructuring, child management skills/parent training and (in-vivo) gradual exposure, and assertiveness training. This is in line with other CBT programs for child anxiety (e.g., Coping Cat) [45]. Children completed homework assignments consisting of gradual exposure under supervision of their parent(s). Multiple RCTs have confirmed efficacy of the Cool Kids Program [2, 46]. The treatment sessions were led by a paid clinical psychologist or a postgraduate psychologist in training under supervision of a clinical psychologist. All clinicians were trained in the use of the Cool Kids program.

### Analytic Strategy

#### Missing Data

Analyses were conducted on an intent-to-treat basis. Missingness patterns were as follows: SCAS-C (T1: 0%; T2: 16.4%; T3: 24.6%), CATS (T1: 0%; T2: 16.4%; T3: 24.6%), interpretation bias (T1: 4.9%; T2: 34.4%), CSR SAD (T1: 11.5%; T2: 14.8%; T3: 31.1%). Little’s MCAR test revealed that the data were missing (completely) at random (*χ*^2^(1114) = 86.40, *p* > 0.999). Missing data were handled using full information maximum likelihood (FIML) within the analysis framework. FIML provides less biased estimates compared to listwise or pairwise deletion [47].

#### Change in Social Anxiety from Pre- to Post-treatment

Three repeated measures (RM) Analysis of Variance (ANOVAs) with a within subjects-factor for time (T1/T2/T3) were conducted to examine within-subjects change over the course of treatment on child- and parent-reported social anxiety symptoms and clinician-rated SAD severity. To correct for multiple testing, results were deemed significant at *p* < 0.0125.

#### Main Analyses

**Fig. 1:**
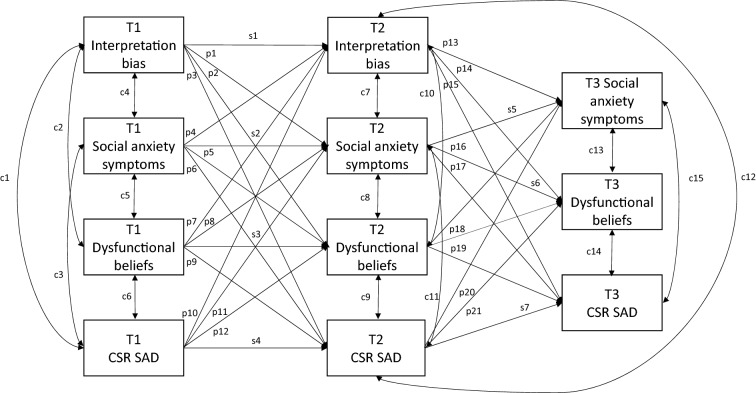
Cross-Lagged Panel Model displaying within-treatment cognitive change from T1 to T2 predicting child-reported and clinician-rated social anxiety severity at T3. Long-term stability coefficients not included for clarity reasons. T1 = pre-treatment assessment; T2 = post-treatment assessment; T3 = 6-month follow-up assessment; T1 social anxiety symptoms = child-reported social anxiety; *CSR SAD* clinician-severity rating social anxiety disorder; *c *covariance; *s* stability coefficient; *p* crosspaths

To examine whether dysfunctional beliefs and interpretation bias show within-subjects change during treatment (aim 1a), two RM ANOVAs were conducted (deemed significant at *p* < 0.0125). Each RM ANOVA included a within subjects-factor for time (T1/T2/[T3]) with interpretation bias or dysfunctional beliefs as the dependent variable. To examine aim 1b and 2, two autoregressive cross-lagged panel models (CLPMs) were conducted. The first CLPM included interpretation bias, dysfunctional beliefs, child-reported social anxiety symptoms (SCAS-C), and clinician-reported SAD severity (SAD CSR) at all timepoints. The second CLPM included the same constructs, but instead of child-reported social anxiety, the parent-reported social anxiety (SCAS-P) was included. CLPMs examine direction of effects while taking extraneous variance into account. Both CLPMs examine whether a change in dysfunctional beliefs and negative interpretation bias over the course of treatment predicted social anxiety symptoms and diagnostic severity at 6-month follow-up (aim 1b), as well as baseline predictors of post-treatment outcome (aim 2). Each model included stability/autoregressive paths (s-paths in Fig. 1) to control for the temporal stability within each variable across time (except for the autoregressive correlation between T2 and T3 interpretation bias), synchronous correlations between variables at each timepoint (c-paths in Fig. 1), and cross-lagged paths between different variables across time (p-paths in Fig. 1).

A robust estimator (MLR) was used to guard against non-normality of the data. Following reporting guidelines, the fit of each model was judged by examining Chi-Square (*p* > 0.05), RMSEA (≤ 0.05) and its associated confidence interval (CI), SRMR (≤ 0.05), CFI (≥ 0.95) [48]. Additionally, a parsimony fit index (PNFI; higher values equal higher parsimony) was reported.^3^ Evidence for temporal precedence was examined by comparing the statistical significance of the cross-lagged paths. By regressing each variable at post-treatment and 6-month follow-up on the score of that variable at the timepoint before (pre- and post-treatment, respectively), the outcome variable in the model was interpreted as the residualized change of that variable. For example, by regressing post-treatment interpretation bias on pre-treatment interpretation bias, post-treatment interpretation bias scores can be interpreted as the residualized change in interpretation bias between these two points [49]. Descriptive analyses and ANOVAs were conducted in SPSS (v.24) [50]. CLPMs were conducted in Rstudio v1.2.1335 (R 3.6.0) [51] with the lavaan package (0.6.5) [52].

## Results

### Change in Social Anxiety

The RM ANOVAs for child-, parent- and clinician-reported social anxiety severity all showed a significant main effect of time. All within-subjects contrasts showed a significant decrease in social anxiety from pre- to post-treatment, and from post-treatment to 6-month follow-up (see Table 2 for statistics and descriptives).

**Table 2:** Mean scores on social anxiety symptoms and cognitive variables on the assessment points

	T1 Pre *M* (*SD*)	T2 Post *M* (*SD*)	T3 Follow-up *M* (*SD*)	Statistics
*Child-report*				
Social anxiety	5.59 (4.14)^a^	3.94 (2.87)^b^	3.54 (2.66)^c^	*F*(1.734,78.051) = 13.24, *p* < .001, *η*^2^ = 0.23
Dysfunctional beliefs	9.26 (10.42)^a^	3.84 (5.33)^b^	3.52 (4.94)^c^	*F*(1.277,57.470) = 15.96, *p* < .001, *η*^2^ = 0.26
Interpretation bias^d^	0.36 (.27)^a^	0.24 (.24)^b^		*F*(1,37) = 7.66, *p* = .009, *η*^2^ = 0.17
Life interference	11.95 (7.94)	7.80 (7.48)	8.02 (7.99)	–
*Parent report*				
Social anxiety	8.84 (3.84)^a^	5.49 (3.02)^b^	4.16 (2.82)^c^	*F*(2,92) = 38.55, *p* < .001, *η*^2^ = 0.46
Life interference	17.36 (7.49)	10.86 (6.73)	10.88 (7.55)	–
*Clinician report*				
CSR SAD	5.69 (0.99)^a^	3.71 (1.32)^b^	3.00 (1.96)^c^	*F*(1.442,54.787) = 48.96, *p* < .001, *η*^2^ = 0.56
CGAS	57.42 (6.65)	70.76 (11.15)	72.40 (16.79)	–

Means with different superscripts within rows are significantly different with all *p*’s < .0125

*CSR SAD* clinician-severity rating social anxiety disorder; *CGAS* Children’s Global Assessment Scale

^d^The mean of person-mean interpretation bias scores are displayed

### Change in Distorted Cognition

The RM ANOVAs indicated a significant effect of time for both dysfunctional beliefs and interpretation bias (Table 2). Within-subjects contrasts indicated a significant decrease in dysfunctional beliefs and interpretation bias from pre- to post-treatment and in dysfunctional beliefs from post-treatment to 6-month follow-up. Overall, these results show that over the course of treatment, both dysfunctional beliefs and interpretation bias decreased. Thus, residualized change scores (see below) represented significant variation from pre- to post-treatment and from post- to 6-month follow-up.

### Cross-Lagged Panel Model Outcomes

**Fig. 2:**
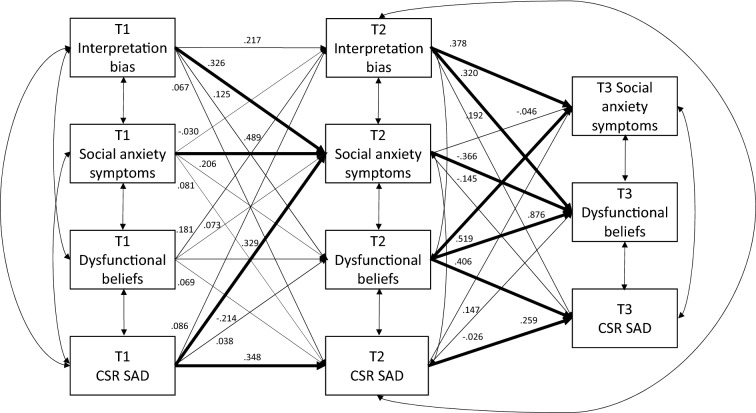
Cross-Lagged Panel Model for child-reported social anxiety including stability and cross-path coefficients. Significant coefficients are indicated by lines in bold. T1 = pre-treatment assessment; T2 = post-treatment assessment; T3 = 6-month follow-up; *CSR SAD* clinician-severity rating social anxiety disorder

The CLPM with child-reported social anxiety symptoms (i.e., child model) showed good fit, *χ*^2^(9) = 9.261, *p* = 0.414, CFI = 0.999, TLI = 0.993, RMSEA = 0.022, CI[0.000, 0.149], SRMR = 0.037, PNFI = 0.158. Table 3 displays all estimates and covariances in the child CLPM and Fig. 2 visualizes these estimates. The second CLPM with parent-reported social anxiety symptoms (i.e., the parent model) did not show a good fit. Two of the indices were unsatisfactory, *χ*^2^(10) = 12.254, *p* = 0.199, CFI = 0.982, TLI = 0.890, RMSEA = 0.074, CI[0.000, 0.153], SRMR = 0.037, PNFI = 0.155. Estimates are not reliable when the fit of the model is not adequate, therefore, they were not interpreted (see Table S1 in the Supplementary Information) [53]. The following sections only refer to the child model.

**Table 3:** Covariances and (un)standardized estimates for all stability- and crosspaths with specifiers in brackets for the CLPM with child-reported social anxiety

	Estimate	*SE*	Standardized estimate	*p*
*Stability paths*				
IB T1 **→** T2 (s1)	0.198	0.106	0.217	0.060
DB T1 **→** T2 (s3)	0.167	0.118	0.329	0.159
DB T2 **→** T3 (s6)	0.827	0.133	0.876	0.000
DB T1 **→** T3	0.117	0.070	0.245	0.094
Soc anx T1 **→** T2 (s2)	0.340	0.101	0.489	0.001
Soc anx T2 → T3 (s5)	− 0.043	0.177	− 0.046	0.810
Soc anx T1 **→** T3	0.073	0.073	0.115	0.317
CSR SAD T1 **→** T2 (s4)	0.468	0.162	0.348	0.004
CSR SAD T2 **→** T3 (s7)	0.442	0.216	0.259	0.041
CSR SAD T1 **→** T3	− 0.194	0.352	− 0.096	0.582
*Cross-lagged paths*				
IB T1 **→** Soc anx T2 (p1)	0.584	0.200	0.326	0.004
IB T1 **→** DB T2 (p2)	0.246	0.254	0.125	0.333
IB T1 **→** CSR SAD T2 (p3)	0.329	0.639	0.067	0.606
DB T1 **→** IB T2 (p7)	0.043	0.047	0.181	0.364
DB T1 **→** Soc anx T2 (p8)	0.034	0.089	0.073	0.705
DB T1 **→** CSR SAD T2 (p9)	0.087	0.256	0.069	0.734
Soc anx T1 **→** IB T2 (p4)	− 0.011	0.065	− 0.030	0.872
Soc anx T1 **→** DB T2 (p5)	0.158	0.152	0.206	0.299
Soc anx T1 **→** CSR SAD T2 (p6)	0.155	0.347	0.081	0.655
CSR SAD T1 **→** IB T2 (p10)	0.022	0.035	0.086	0.535
CSR SAD T1 **→** DB T2 (p12)	0.020	0.060	0.038	0.737
CSR SAD T1 **→** Soc anx T2 (p11)	− 0.105	0.048	− 0.214	0.027
IB T2 **→** Soc anx T3 (p13)	0.684	0.252	0.378	0.007
IB T2 **→** DB T3 (p14)	0.654	0.279	0.320	0.019
IB T2 **→** CSR SAD T3 (p15)	10.546	10.434	0.192	0.281
DB T2 **→** Soc anx T3 (p18)	0.434	0.108	0.519	0.000
DB T2 **→** CSR SAD T3 (p19)	10.513	0.739	0.406	0.041
CSR SAD T2 **→** DB T3 (p21)	− 0.010	0.030	− 0.026	0.742
CSR SAD T2 **→** Soc anx T3 (p20)	0.050	0.037	0.147	0.179
Soc anx T2 **→** DB T3 (p16)	− 0.381	0.167	− 0.366	0.022
Soc anx T2 **→** CSR SAD T3 (p17)	− 0.597	10.033	− 0.145	0.563
*Covariances*				
IB T1 ~~ DB T1 (c2)	0.072	0.033	0.264	0.028
IB T1 ~~ Soc anx T1 (c4)	0.072	0.023	0.395	0.002
IB T1 ~~ CSR SAD T1 (c1)	0.003	0.037	0.010	0.945
DB T1 ~~ CSR SAD T1 (c6)	0.191	0.111	0.190	0.085
Soc anx T1 ~~ DB T1 (c5)	0.520	0.095	0.736	0.000
Soc anx T1 ~~ CSR SAD T1 (c3)	0.135	0.082	0.204	0.100
Soc anx T2 ~~ DB T2 (c8)	0.094	0.026	0.667	0.000
Soc anx T2 ~~ CSR SAD T2 (c11)	0.117	0.070	0.306	0.093
Soc anx T2 ~~ IB T2 (c7)	0.002	0.012	0.023	0.888
IB T2 ~~ DB T2 (c10)	− 0.019	0.015	− 0.190	0.195
IB T2 ~~ CSR SAD T2 (c12)	− 0.001	0.050	− 0.004	0.984
DB T2 ~~ CSR SAD T2 (c9)	0.057	0.112	0.111	0.610
Soc anx T3 ~~ DB T3 (c13)	0.008	0.013	0.097	0.549
Soc anx T3 ~~ CSR SAD T3 (c15)	0.105	0.083	0.203	0.205
DB T3 ~~ CSR SAD T3 (c14)	0.145	0.075	0.323	0.053

*IB* interpretation bias; *DB* dysfunctional beliefs; *Soc anx* social anxiety; *CSR SAD* clinician-severity rating social anxiety disorder

### Predicting 6-Month Follow-Up Outcome with Pre- to Post-treatment Cognitive Change

The cross-path coefficients indicated that greater pre- to post-treatment changes in interpretation bias significantly predicted greater change in child-reported social anxiety symptoms, but not clinician-rated SAD severity, in the period from post-treatment to 6-month follow-up (see Table 3 for all estimates). Greater pre- to post-treatment changes in dysfunctional beliefs predicted significantly greater change in both child-reported social anxiety and clinician-rated SAD severity at 6-month follow-up. Hence, children who showed greater change in interpretation bias and dysfunctional beliefs from pre- to post-treatment, also self-reported greater change in social anxiety symptoms at 6-month follow-up. In addition, children who self-reported greater decreases in dysfunctional beliefs at post-treatment, also exhibited greater change in social anxiety severity at 6-month follow-up as reported by the clinician. Interestingly, greater pre- to post-treatment change in child-reported social anxiety predicted less change in dysfunctional beliefs in the period between post-treatment and 6 months follow-up. Indicating that children who reported more change in social anxiety symptoms during treatment, reported less change in dysfunctional beliefs from post- to 6-month follow-up. There was no significant effect of pre- to post-treatment changes in clinician-rated SAD severity on dysfunctional beliefs at 6-month follow-up. Thus, these results indicate that there is a bidirectional effect of child self-reported social anxiety symptoms on dysfunctional beliefs, whereas no bidirectional effect of clinician-rated SAD severity on dysfunctional beliefs was found.

### Pre-treatment Cognitive Predictors of Post-treatment Outcome

Cross-paths from pre- to post-treatment showed that interpretation bias at pre-treatment significantly predicted child-reported social anxiety at post-treatment. This indicates that children who had higher interpretation bias levels before treatment, reported greater changes in child-reported social anxiety levels at post-treatment (see Table 3 for all estimates). Pre-treatment dysfunctional belief levels did not significantly predict changes at post-treatment in child- or clinician-reported social anxiety.

## Discussion

In the current study, a clinical sample of children with SAD received CBT in a longitudinal, single condition treatment-design. The first goal was to examine how treatment works by examining (a) whether children with SAD showed pre- to post-treatment changes in distorted cognition (i.e., interpretation bias for social scenarios and dysfunctional social beliefs), and (b) whether changes in distorted cognition during treatment predicted social anxiety outcomes at 6-month follow-up. The second goal was to examine for whom treatment works, by examining whether baseline levels of cognitive distortions predict changes in social anxiety symptoms at post-treatment. This preliminary study is the first to focus on potential cognitive mediators and predictors of CBT outcome in childhood SAD, a population that would greatly benefit from treatment improvements.

In line with our first hypothesis that distorted cognition should change during treatment, results showed pre- to post-treatment decreases in both interpretation bias and dysfunctional beliefs. It is important to point out that, in the absence of a control condition not receiving treatment, it is not possible to attribute these changes to treatment effects. Nonetheless, these results are consistent with the underlying assumption of CBT-models that distorted cognition is an important target of treatment and should decrease over its course [7–9, 20]. This study shows that SAD-specific cognitions changed during treatment and hereby extends previous studies showing that distorted cognition changes during CBT in mixed anxiety disorder groups [16, 17, 24].

The findings showed partial support for the second hypothesis that decreases in distorted cognition would predict social anxiety at 6-month follow-up. Pre- to post-treatment changes in interpretation bias and dysfunctional beliefs were significantly and prospectively related to changes in child-reported social anxiety at 6-month follow-up. Thus, children who showed more change (i.e., decreases) in distorted cognition, showed a greater decrease in social anxiety 6 months after treatment. This is in line with previous studies on children with a mix of anxiety disorders, finding an association between changes in general negative cognition during CBT and anxiety symptom change [23]. The results are also in line with studies pointing to a mediating role for distorted cognition in anxiety symptom change in children with heterogenous anxiety disorders [15, 54]. The current study extends these findings to children with SAD and SAD-specific distorted cognition. The results are, however, not in line with previous studies that failed to find that changes in dysfunctional beliefs predicted anxiety reduction [16, 25]. A change in dysfunctional beliefs may be particularly related to social anxiety symptom change rather than general anxiety symptoms (e.g., total scale sum scores).

Importantly, greater changes in social anxiety symptoms from pre- to post-treatment predicted less changes in dysfunctional beliefs at 6-month follow-up. Thus, although changes in social anxiety symptoms from pre- to post-treatment change also have an effect on changes in dysfunctional beliefs, this effect is negative. This may indicate that those children showing greater changes in social anxiety at post-treatment may have already had larger changes in their dysfunctional beliefs at post-treatment as well. Hence, there may have been less room left for change from post-treatment to 6-month follow-up in dysfunctional beliefs. These results may indirectly and carefully suggest a directional effect of changes in dysfunctional beliefs preceding anxiety changes such that reductions in dysfunctional beliefs precede reductions in social anxiety symptoms, but not the other way around. This would be in line with assumptions from cognitive models that higher dysfunctional beliefs contribute to anxiety [7]. However, future studies with more assessment points during both the treatment and follow-up period are necessary to show that within-treatment changes in dysfunctional beliefs occur before changes in social anxiety changes occur to make this claim.

Interestingly, changes in distorted cognition during treatment were differentially related to 6-month follow-up on child- and clinician-reported outcomes. Changes in dysfunctional beliefs from pre- to post-treatment predicted social anxiety severity per child- and clinician-report at 6-month follow-up. However, changes in interpretation bias from pre- to post-treatment predicted only child-reported social anxiety levels at 6-month follow-up. A possible explanation may be that the ADIS-IV SAD section explicitly addresses a list of dysfunctional beliefs during the assessment which may directly reflect in clinician-rated SAD severity (CSR). In contrast, interpretation bias for ambiguous situations may not be as explicitly addressed. This stresses the merit of examining distorted cognition with different measures tapping into different stages of the information-processing pathway for adequate treatment evaluation. Furthermore, this shows the complexity of differential associations between informant reports on anxiety and cognitive measures throughout different treatment-stages.

The findings of this study may provide an explanation for the mixed findings in previous studies where a large variety of informant reports and cognitive measures was employed [18, 23, 24]. For example, the parent-model did not fit the data. This is in line with previous studies showing that the association between cognitive change and treatment outcomes was only found for child-report and not for parent-report [26, 27]. Taken together, these findings suggest that changes in child-reported distorted cognition do not explain much variance in parent-reported social anxiety symptoms. Given that distorted cognition is an internal process, parents may not be aware of children’s distorted cognition but rather report on more visible behavioural and affective symptoms, decreasing the shared variance on reports of cognitive aspects on both measures. In general, this study supports previous findings showing an association between decreases in distorted cognition during CBT and treatment gains and extends these results to a treatment-seeking SAD sample. Furthermore, this study adds to these studies by showing the long-term durability of the prospective relation between cognitive change and treatment gains [17–19, 23, 26, 27]. Clearly, however, more studies are needed to replicate the current results and to address other potential (cognitive) mediators of treatment for childhood SAD.

With regards to our third exploratory hypothesis, results showed that children with higher interpretation bias-levels at baseline showed greater changes in self-reported social anxiety symptoms at post-treatment. This result indicates that children who started treatment with a stronger tendency to interpret social events as negative, benefitted more from treatment than those who had lower interpretation bias levels. This is in line with a previous study by Waters et al. [22] that found that anxious children with higher negative attention bias levels at pre-treatment showed significantly more improvement in their anxiety symptoms after treatment. Children in general showed a decrease in their interpretation bias from pre- to post-treatment, which may reflect regression towards the mean. Alternatively, it may imply treatment-associated changes in interpretation style when encountering ambiguous social situations. Dysfunctional belief levels at baseline did not predict post-treatment social anxiety. This shows that it is important to account for baseline differences in interpretation bias in predicting which children with SAD will respond to treatment. These results again also underline the importance of including multiple distorted cognition indices, such as attention bias, interpretation bias and memory bias, as they may differentially predict changes over time. As this is the first examination of cognitive treatment predictors in children with SAD, further studies with appropriate control conditions are necessary to elucidate which cognitive factors relate to treatment outcome.

The current study had both strengths and limitations. A strength of this study was the inclusion of multiple informant reports allowing us to compare different perspectives on the status of the child’s anxiety symptoms throughout treatment. Second, two distorted cognition indices were included to examine possible differences in changes during CBT and in predictive value for 6-month follow-up treatment outcomes. Results showed that this approach has merit, as both baseline prediction and changes over the course of treatment were found and temporal precedence of theorized cognitive mechanisms may not be similarly present for all cognitive variables. A third strength was the inclusion of a 6-month follow-up assessment, which allowed examination of durability of changes over time.

A first limitation of this study is that, in the absence of a control condition, observed changes could be the result of the passing of time or other variables. However, research suggests that anxiety symptoms rarely remit without intervention and SAD in particular is one of the most chronic of the anxiety disorders [55, 56]. Second, mediation test conditions were not fully present. An in-treatment assessment would have enabled us to establish whether temporal precedence was present pre- to post-treatment, allowing for more stringent conclusions on treatment mechanisms. Future research should include longitudinal designs with one or more within-treatment assessments to elucidate temporal precedence of changes in distorted cognition and social anxiety. Third, this study included children with SAD in their diagnostic profile. This means that not all children had a primary SAD. Although this could be considered a limitation, research has shown that the presence of SAD anywhere in the diagnostic profile are more likely than children without SAD in their profile to continue to meet diagnostic criteria and to show residual anxiety symptoms following treatment [2–4]. As these children have shown distorted cognition specific to SAD to be diagnosed as such, this sample composition gives insights to SAD-specific distorted cognition.

A fourth limitation was the lack of an interpretation bias-assessment at 6-month follow-up. This did not allow examination of possible bidirectional effects of social anxiety levels on interpretation bias, nor did it account for the multilevel nature of the data [57]. Consequently, these results should be interpreted carefully and await future research efforts. Fourth, a relatively small sample was included which leads to increased standard errors. We were dependent on the number of children with social anxiety disorder presenting to the clinic for treatment. We have selected all children that came in during the study period that met our inclusion criteria. A larger sample size may have led to some of the stability paths reaching significance due to increased statistical power. This also prevented the inclusion of pre- to 6-month follow-up predictors and of covariates that may have been of interest (e.g., depression, age). Depressive symptoms and possible associations with age, even in this younger age range, should be considered in the design of future studies. Fifth, our sample did not allow for a comparison between children with and without SAD. A follow-up study comparing cognitive change and the link to treatment outcomes between children with and without SAD could elucidate whether cognitive change differs between these groups. Finally, although this study addressed two types of cognitions that are considered to be crucial for anxiety maintenance and treatment, they did not explain all the variance in treatment gains at 6-month follow-up. Other theorized mechanisms underlying CBT’s effectiveness such as perceived coping ability and (behavioural) avoidance should also be prioritized, or tested in parallel, in future research (Spence & Rapee, 2016). This should facilitate a broader view on working mechanisms of CBT in childhood SAD.

Despite its limitations, the current study represents an important initial attempt to investigate *how* and for *whom* treatment works in a sample of children with SAD. Results suggested that children who showed greater decreases in their distorted cognitions at post-treatment, show better maintenance of treatment gains at 6-month follow-up. However, the other side of the coin is that children who show less improvement in their distorted cognitions, may benefit less from treatment.

## Summary

Children with SAD show the greatest risk of not benefiting from CBT, but it is unknown how to improve their outcomes [4]. The current study is an important initial step to investigate *how* and for *whom* treatment works in a sample of children with SAD. A clinical sample of children with SAD received CBT in a longitudinal, single condition treatment-design. Results from a cross-lagged panel model suggested that children who showed greater decreases in their distorted cognitions at post-treatment, show better maintenance of treatment gains at 6-month follow-up. However, children who show less improvement in their distorted cognitions, may benefit less from treatment. CBT for child anxiety is often provided in a generic, one-size-fits-all format. CBT in this format reduces distorted social cognitions, but for children who do not show cognitive change, treatment outcome is less favourable. These children may require additional efforts to reduce their distorted cognitions in order to benefit from treatment. Disorder-specific SAD treatment programs that incorporate alternative ways of targeting disorder-specific cognitions (e.g., video-feedback) show promise [58, 59]. This study provides an important first step by providing preliminary evidence that an improved focus on disorder-specific distorted cognition may facilitate treatment outcomes for childhood SAD.

## Supplementary Information

Below is the link to the electronic supplementary material.

Supplementary file1 (DOCX 24 kb)

## Footnotes

1 To examine possible carry-over effects of the CBM-I on treatment outcome, a one-way Analysis of Variance (training group: neutral, positive) was conducted. There were no carry-over effects of the CBM-I, as evidenced by non-significant effects of the training on SCAS-C/P-scores (total and social anxiety subscale), primary and secondary disorder severity and CGAS for child-report and parent-report (post- and 6-month follow-up assessment; all *p*’s > 0.05). Age and sex were unrelated to child-reported social anxiety (*r* = 0.22, *p* = 0.087; *r* = 0.22, *p* = 0.086), CSR SAD (*r* = − 0.25, *p* = 0.070; *r* = 0.15, *p* = 0.275), interpretation bias (*r* = 0.22, *p* = 0.099; *r* = 0.03, *p* = 0.824), and dysfunctional beliefs (*r* = 0.15, *p* = 0.246; *r* = 0.01, *p* = 0.913). Therefore, training group, age and sex were not included in the main analyses.

2 For the full procedure, see Klein et al. [30].

3 As there are no recommended cut-off guidelines for parsimony, this index was included for informative reasons only [48].
