# Supplementary material for: The Role of Distorted Cognitions in Mediating Treatment Outcome in Children with Social Anxiety Disorder: A Preliminary Study
Source: Child Psychiatry Hum Dev. 2021 Oct 21;54(2):558–69. doi: 10.1007/s10578-021-01268-6 (PMC9977708; doi:10.1007/s10578-021-01268-6)
Supplement: Supplementary file 1 — Supplementary file1 (DOCX 24 kb) [file 10578_2021_1268_MOESM1_ESM.docx]

The Role of Distorted Cognitions in Mediating Treatment Outcome in Children with Social Anxiety Disorder: A Preliminary Study

**Supplemental Information**

**Table S1**

Lynn Mobach, MSc^*^

Ronald M. Rapee, Ph.D

Anke M. Klein, Ph.D

Lynn Mobach, Department of Clinical Psychology, Behavioural Science Institute, Radboud University, the Netherlands; Centre for Emotional Health, Department of Psychology, Macquarie University, Australia, https://orcid.org/0000-0002-0172-8525; Ronald M. Rapee, Centre for Emotional Health, Department of Psychology, Macquarie University, Australia, https://orcid.org/0000-0002-1724-1076; Anke M. Klein, Institute of Psychology, Unit Developmental and Educational Psychology, Leiden University, the Netherlands

*Correspondence to: Lynn Mobach, Centre for Emotional Health, Department of Psychology, Macquarie University, Sydney, NSW, 2109, Australia.

Present address: Behavioural Science Institute, Department of Clinical Psychology, Radboud University, Montessorilaan 3, 6525 HR, Nijmegen, the Netherlands; e-mail: [l.mobach@psych.ru.nl](mailto:l.mobach@psych.ru.nl), +31682285599

**Table S1**

Table 1.

*Covariances and (un)standardized estimates for all stability- and crosspaths for the parent model with specifiers in brackets*.

|  | Estimate | *SE* | Standardized estimate | *p* |
| --- | --- | --- | --- | --- |
| Stability paths |  |  |  |  |
| IB^a^ T1 🡪 T2 (s1) | .231 | .115 | .257 | .044 |
| DB^b^ T1 🡪 T2 (s3) | .233 | .088 | .458 | .008 |
| DB T2 🡪 T3 (s6) | .487 | .107 | .515 | .000 |
| DB T1 🡪 T3 | .088 | .066 | .182 | .181 |
| Soc anx^c^ T1 🡪 T2 (s2) | .356 | .100 | .444 | .000 |
| Soc anx T2 🡪 T3 (s5) | .281 | .144 | .229 | .052 |
| Soc anx T1 🡪 T3 | .509 | .100 | .517 | .000 |
| CSR SoAD^d^ T1 🡪 T2 (s4) | .522 | .155 | .389 | .001 |
| CSR SoAD T2 🡪 T3 (s7) | .261 | .234 | .178 | .261 |
| CSR SoAD T1 🡪 T3 | -.024 | .336 | -.012 | .944 |
| Cross-Lagged Paths |  |  |  |  |
| IB T1 🡪 Soc anx T2 (p1) | .104 | .244 | .054 | .671 |
| IB T1 🡪 DB T2 (p2) | .332 | .224 | .168 | .138 |
| IB T1 🡪 CSR SoAD T2 (p3) | .338 | .583 | .068 | .562 |
| DB T1 🡪 IB T2 (p7) | .020 | .039 | .087 | .606 |
| DB T1 🡪 Soc anx T2 (p8) | .119 | .066 | .243 | .069 |
| DB T1 🡪 CSR SoAD T2 (p9) | .108 | .189 | .085 | .569 |
| Soc anx T1 🡪 IB T2 (p4) | .076 | .071 | .200 | .289 |
| Soc anx T1 🡪 DB T2 (p5) | .021 | .094 | .025 | .825 |
| Soc anx T1 🡪 CSR SoAD T2 (p6) | .137 | .266 | .066 | .606 |
| CSR SoAD T1 🡪 IB T2 (p10) | .016 | .033 | .064 | .639 |
| CSR SoAD T1 🡪 DB T2 (p12) | .018 | .074 | .034 | .809 |
| CSR SoAD T1 🡪 Soc anx T2 (p11) | -.062 | .081 | -.119 | .443 |
| IB T2 🡪 Soc anx T3 (p13) | -.198 | .322 | -.076 | .539 |
| IB T2 🡪 DB T3 (p14) | .374 | .250 | .180 | .135 |
| IB T2 🡪 CSR SoAD T3 (p15) | .948 | 1.215 | .118 | .435 |
| DB T2 🡪 Soc anx T3 (p18) | .200 | .161 | .168 | .214 |
| DB T2 🡪 CSR SoAD T3 (p19) | .539 | .462 | .147 | .243 |
| CSR SoAD T2 🡪 DB T3 (p21) | -.021 | .030 | -.057 | .470 |
| CSR SoAD T2 🡪 Soc anx T3 (p20) | .038 | .052 | .080 | .467 |
| Soc anx T2 🡪 DB T3 (p16) | .264 | .090 | .270 | .004 |
| Soc anx T2 🡪 CSR SoAD T3 (p17) | .911 | .748 | .241 | .223 |
| Covariances |  |  |  |  |
| IB T1 ~~ DB T1 (c2) | .076 | .033 | .278 | .020 |
| IB T1 ~~ Soc anx T1 (c4) | .051 | .022 | .304 | .017 |
| IB T1 ~~ CSR SoAD T1 (c1) | .014 | .039 | .055 | .711 |
| DB T1 ~~ CSR SoAD T1 (c6) | .183 | .110 | .181 | .096 |
| Soc anx T1 ~~ DB T1 (c5) | .254 | .082 | .387 | .002 |
| Soc anx T1 ~~ CSR SoAD T1 (c3) | .163 | .085 | .262 | .054 |
| Soc anx T2 ~~ DB T2 (c8) | .062 | .023 | .339 | .007 |
| Soc anx T2 ~~ CSR SoAD T2 (c11) | .186 | .059 | .385 | .002 |
| Soc anx T2 ~~ IB T2 (c7) | .016 | .014 | .175 | .247 |
| IB T2 ~~ DB T2 (c10) | -.016 | .012 | -.167 | .205 |
| IB T2 ~~ CSR SoAD T2 (c12) | -.026 | .051 | -.101 | .612 |
| DB T2 ~~ CSR SoAD T2 (c9) | .080 | .108 | .155 | .459 |
| Soc anx T3 ~~ DB T3 (c13) | .017 | .016 | .156 | .268 |
| Soc anx T3 ~~ CSR SoAD T3 (c15) | .392 | .107 | .562 | .000 |
| DB T3 ~~ CSR SoAD T3 (c14) | .114 | .089 | .249 | .200 |

*Note.* ^a^ IB = Interpretation bias; ^b^ DB = Dysfunctional beliefs; ^c^ Soc anx = Social anxiety as reported by the parent; ^d^ CSR SoAD = Clinician-severity rating social anxiety disorder
